# Complex Dysautonomia in a Patient With Cerebral Cavernous Malformations Due to a KRIT1 Pleiotropic Gene Mutation

**DOI:** 10.7759/cureus.55202

**Published:** 2024-02-29

**Authors:** Roel Janssen, Maxime Ariëns, Jessie van Genugten, Linda Jacobi, Ger Koek

**Affiliations:** 1 Faculty of Health, Medicine, and Life Sciences, Maastricht University, Maastricht, NLD; 2 Department of Primary Care Medicine, Radboud University Medical Center, Nijmegen, NLD; 3 Department of Neurology, Ziekenhuisgroep Twente Hengelo, Hengelo, NLD; 4 Department of Radiology and Nuclear Medicine, Maastricht University Medical Centre, Maastricht, NLD; 5 Department of Internal Medicine, Division of Gastroenterology and Hepatology, Maastricht University Medical Centre, Maastricht, NLD

**Keywords:** vagal dysfunction, vagal nerve, barrington’s nucleus, pons, cavernous malformation, autonomic dysfunction, dysautonomia

## Abstract

Dysautonomia is a disruption of the body's autonomic processes. Symptoms vary among patients, depending on the underlying disease pathways. Given that symptoms can affect all organ functions, dysautonomia often significantly impacts quality of life. However, due to its complex and varied presentation, early recognition of dysautonomia remains a challenge, yet it is crucial for improving patient outcomes. We report a case of a patient with a KRIT1 mutation presenting with dysautonomia causing urological, sexual, and bowel dysfunction. We hypothesize that the patient's symptoms are due to a pontine cavernous malformation (CM) caused by the KRIT1 mutation. A literature review was conducted to establish a link between pontine CM and dysautonomia.

## Introduction

Dysautonomia is a dysfunction of the autonomic nervous system that can negatively impact health [1]. It is often left unrecognised by clinicians, leading to delayed treatment and reduced quality of life for patients [1]. Dysautonomia can have various aetiologies, including degenerative neurological diseases such as Parkinson's disease and systemic illnesses like diabetes mellitus, as well as iatrogenic causes [2]. Symptoms may include postural hypotension, urological, sexual, and bowel dysfunction, and sudoresis, but they can vary depending on the underlying disease [2]. This paper presents a patient with dysautonomia-related symptoms caused by multiple cerebral cavernous malformations (CCMs), resulting from an autosomal dominant KRIT1 (CCM1) gene mutation. CCMs are clusters of intracranial vascular malformations that carry an increased risk of bleeding [3]. We hypothesise that the patient's dysautonomia is secondary to the CCM found in the pons.

## Case presentation

A 63-year-old Caucasian male has experienced dysautonomic symptoms intermittently since the age of 20, which included urological issues (dysuria, altered urinary frequency, nocturia, and recurrent urinary tract infections due to urinary retention) and sexual issues (erectile dysfunction and problems with ejaculation). The cause of these dysautonomic symptoms remained undiagnosed. Over the years, these symptoms were accompanied by gastric dumping, irritable bowel syndrome, and non-alcoholic fatty liver disease (NAFLD), diagnosed via liver ultrasound. The patient's weight was noted to be in the overweight range. He has no history of medication use.

**Figure 1:**
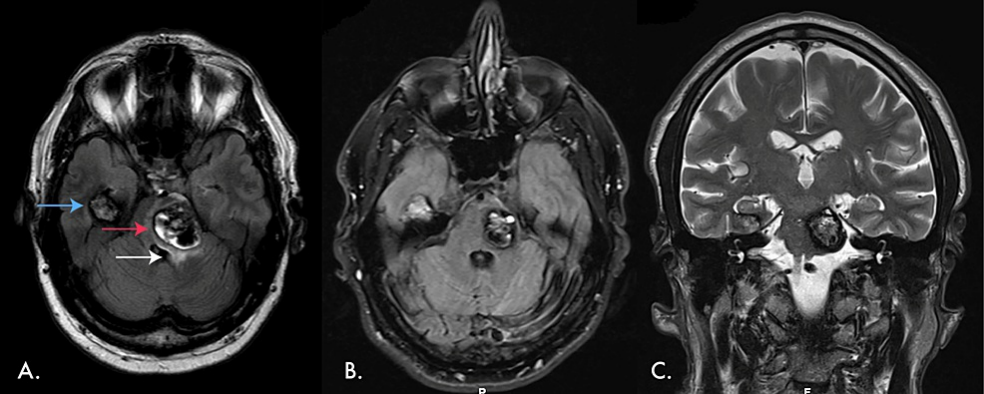
MR scan of the brain showing the pontine CM A: T2-weighted MR scan of the brain at presentation, showing the CM located in the pons (red arrow) and in the hyperacute bleeding phase with oedema (white arrow). The CM in the temporal lobe is also visible (blue arrow). B and C: T2-weighted MR scan of the brain, showing an axial and coronal view of the pontine CM in the non-bleeding phase. MR: magnetic resonance, CM: cavernous malformation

Decades later, at age 63, he presented with new-onset neurological symptoms: impaired gait, double vision, and sensation disorders on the right side of his body. An MRI investigation revealed multiple CCMs, with the three largest located in the left frontal lobe, right temporal lobe, and pontine region. The pontine cavernous malformation (CM), measuring 21 millimetres in diameter, showed signs of intermittent bleeding, suggesting recent activity (Figure 1). In addition to cerebral manifestations, the patient exhibited CM-derived cutaneous lesions on the left leg and abdominal wall, as well as CCMs in the liver and kidney.

**Figure 2:**
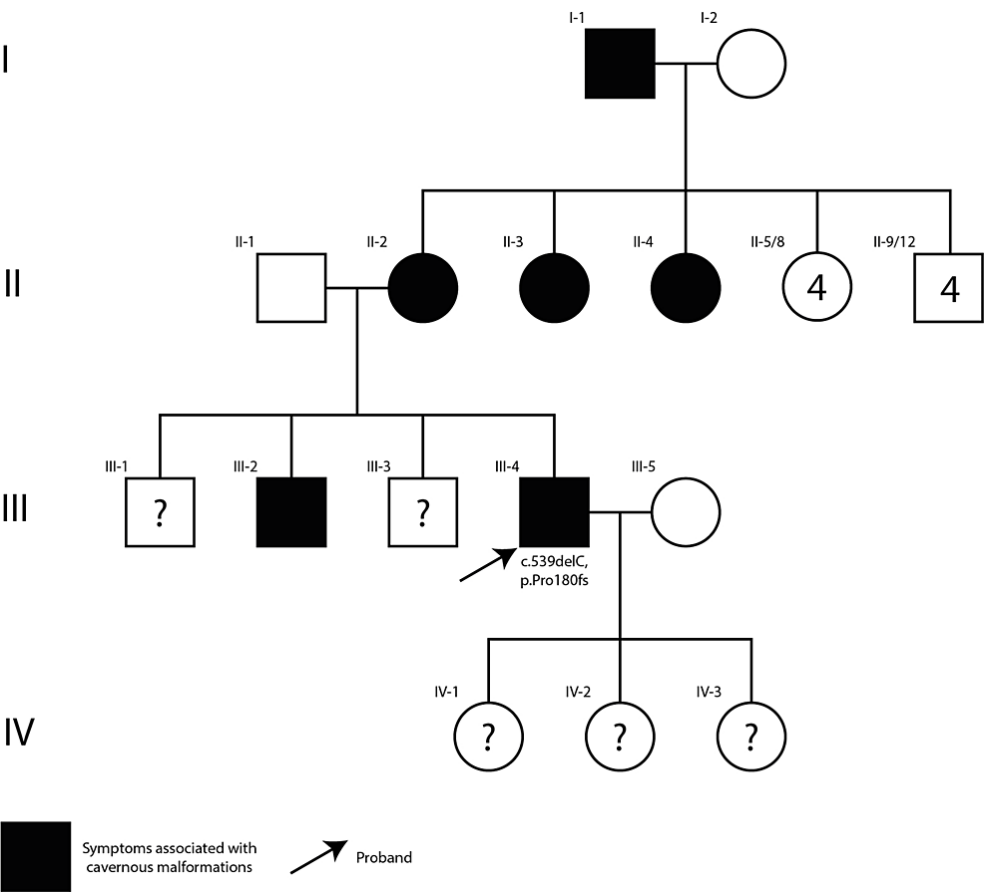
Pedigree of the patient The patient (proband) is indicated by the black arrow

Genetic testing revealed a c.539delC, p.Pro180fs mutation in the KRIT1 gene, indicative of a frameshift mutation leading to a truncated protein that disrupts vascular integrity. This genetic abnormality is implicated in the multifocal nature of his CCMs. The patient's family history, illustrated in Figure 2, reveals a pattern of symptoms and conditions related to CMs (including cutaneous findings and neurological symptoms) among relatives, suggesting a hereditary predisposition to the mutation.

The patient undergoes annual follow-up visits at the neurology, urology, and gastroenterology outpatient clinics. No specific treatment for the CCMs has been adopted; he has chosen not to use antiepileptic drugs due to their side effects. Neurosurgical intervention was considered at various centres but was deemed infeasible due to the malformation's location and the high risk of severe complications.

## Discussion

We hypothesise that the patient's fluctuating symptoms result from intermittent pontine CM haemorrhages. CCMs are prone to hemorrhagic events, which can compress and impair the surrounding areas [3]. Given that those critical nuclei of the autonomic nervous system are in the brainstem [4], we deduce that the bleeding of the pontine CM is responsible for dysautonomia. In the following section, we will explain this deduction by considering the anatomical characteristics of the CM and the surrounding nuclei and analysing the patient's symptoms to the location of the CM.

Anatomical characteristics

The pontine CM is located in the pons, which is abundant with autonomic structures that regulate autonomic functions. Regarding the symptoms that our patient has, we hypothesise that the most critical pontine structures involved are (i) Barrington's nucleus, also known as the pontine micturition centre or pelvic organ stimulating centre, located in the dorsomedial pontine tegmentum [5] and contributing to micturition and pelvic and colon activity [6,7]; (ii) the L-region, also known as the pontine storage centre or pelvic floor stimulating centre [5,8], located in the ventral tegmentum [5] and regulating urine storage [7] and ejaculation [8]; and (iii) the dorsal longitudinal fasciculus and the parabrachial nucleus, both involved in conveying visceral information to various brain structures [9]. These nuclei may be compressed or damaged by the periodic bleeding or dilation of the pontine CM [10].

Although we have found evidence of one bleeding episode, it is reasonable to deduce that our patient is more prone to recurrent haemorrhages due to the pontine location of the CM, the large size of the lesion, and his developmental venous anomaly resulting from the KRIT1 mutation [10]. The intermittent nature of his symptoms coincides with the natural history of CCMs, where changes in the size of the malformation due to active bleeding or resolution of bleeding may potentially exert different levels of pressure on the surrounding pontine structures [11]. The dysautonomia symptoms likely occur during acute CCM bleeding and recede during the resolution of the haemorrhage [10].

Urological symptoms

**Figure 3:**
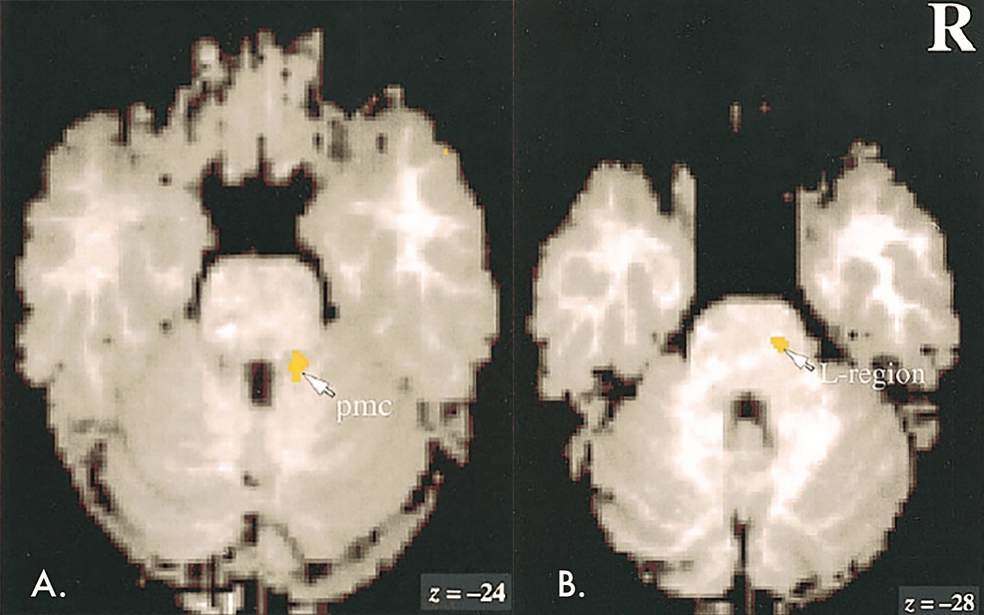
PET-CT of the brain, showing the pontine micturition center and L-region, located in the pons PMC: pontine micturition center; PET-CT: positron emission tomography-computed tomography Published with permission: Blok et al. (1997) [5]

The proximity of the pontine CM to Barrington's nucleus and L-region (Figure 3) may explain the patient's urological symptoms, as these regions are known to be involved in the micturition process [7]. Barrington's nucleus receives input from the bladder regarding fullness through pelvic nerves and the periaqueductal grey [7], and its stimulation promotes micturition by increasing parasympathetic activity in the bladder and decreasing the sympathetic tone [7]. The L-region, located ventrolateral to the PMC, increases activity in the external urethral sphincter's striated muscle via the pudendal nerves, promoting continence [7]. However, the L-region has mainly been identified in animal models, and research on its existence in humans is limited. Blok et al. [5] identified an area ventrolateral to the Barrington's nucleus in healthy volunteers that may be the human L-region based on its anatomical and functional similarities to animal studies (Figure 3). This human L-region may be indirectly supported by the observation that patients with brain stem injuries often have altered continence [12]. By comparing the PET-CT study images of Blok et al. [5] (Figure 3) with the MR images of the patient, it was deduced that the pontine CM may have affected both the Barrington's nucleus and L-region in the case of hyperacute bleeding. The resulting oedema infiltrates an area of the pons that encompasses both nuclei due to the proximity of the CCM to Barrington's nucleus and L-region, disrupting the signalling pathways of both nuclei.

Sexual dysfunction

The patient's sexual dysfunction can be explained by the disruption of Barrington's nucleus and L-region, which are both involved in sexual functions [8,13] in normal conditions. The neurons of these structures terminate on parasympathetic motoneurons in the sacral segment of the spinal cord (Onuf's nucleus) [8,13], which innervate the ischiocavernosus and bulbospongiosus muscles via the deep branch of the perineal nerve, both of which play a role in ejaculation by propelling semen through the bulbar and penile urethra for ejection via the meatus [8,13]. In addition, Barrington's nucleus is also involved in penile erection [14]. These considerations support the hypothesis that the pontine bleeding detected in the patient may have induced sexual dysfunction by altering the function of Barrington's nucleus and L-region. This is not surprising, as literature has already shown that brain stem lesions due to vascular or neurodegenerative diseases may cause erectile dysfunction [15].

Gastrointestinal symptoms

The gastric dumping and increased intestinal motility experienced by the patient may be related to altered neural signalling in the pons, potentially involving structures such as the dorsal longitudinal fasciculus and the parabrachial nucleus [9,16]. The brain stem controls gastrointestinal function through afferent and efferent projections of the vagal nerve [17]. Vagal afferent neurons transmit information from the stomach to the nucleus tractus solitaries (NTS) neurons, passing the signal on to higher brain centres such as the hypothalamus's paraventricular nucleus [18]. The NTS communicates with the hypothalamus through the dorsal longitudinal fasciculus and parabrachial nucleus in the pons [9,16], and the signal is also relayed to the neurons of the dorsal motor nucleus of the vagus (DMV) to facilitate the vagovagal reflex [18]. The hypothalamus integrates this information and sends efferent fibres to the DMV to control gastrointestinal parasympathetic activity [18]. The dorsal longitudinal fasciculus and parabrachial nucleus mediate the interaction between the hypothalamus and the DMV [9,16]. Increased parasympathetic activity increases gastrointestinal motility and gastric emptying [18]. Therefore, it is reasonable to deduce that the CM in the pons affects both afferent and efferent pathways by disturbing the dorsal longitudinal fasciculus and parabrachial nucleus, potentially causing the patient's gastrointestinal symptoms.

Furthermore, the CM in the pons may contribute to the development and progression of the patient's NAFLD. The liver is innervated by the vagal and spinal nerves for parasympathetic and sympathetic signals, respectively [19]. Since the parasympathetic innervation follows the same pathway described above for the stomach [9,16,18,19], the disruption of the dorsal longitudinal fasciculus and parabrachial nucleus by the pontine CM may also result in parasympathetic denervation of the liver. The imbalance between sympathetic and parasympathetic activity caused by this disruption may result in enhanced sympathetic input, increasing lipolysis, and releasing free fatty acids from adipose tissue and their deposits in the liver [20]. This can contribute to the onset and worsening of NAFLD [20].

## Conclusions

Dysautonomia is characterised by changes in autonomic nervous system function that can negatively impact multiple bodily systems. We presented a case of dysautonomia caused by a CCM in the pons, a region essential for autonomic function. This dysautonomia has adversely affected the patient's urological, sexual, and gastrointestinal autonomic functions. Adopting a holistic approach to diagnosing dysautonomia is necessary, as its presence can be challenging to interpret and recognise as a single disorder. Further understanding of the mechanisms underlying dysautonomia will provide new insights into treatment options that can improve patient health and quality of life.
